# Knowledge, beliefs and communication preferences with regards to the HPV vaccine; the perspective of unvaccinated Greek adolescent girls, young women, and mothers of vaccine-eligible girls

**DOI:** 10.1080/21642850.2018.1505518

**Published:** 2018-08-02

**Authors:** Christina Karamanidou, Κostas Dimopoulos

**Affiliations:** a Department of Social and Educational Policy, Faculty of Social Sciences, University of Peloponnese, Damaskinou and Kolokotroni str, Korinthos, Greece; b Department of Education, University of Nicosia, Nicosia, Cyprus

**Keywords:** HPV vaccine, knowledge, beliefs about medicines, health communication

## Abstract

**Background:** Every year in Europe 60,000 women develop cervical cancer and 30,000 die from the disease. HPV vaccines are currently believed to constitute an important element of cervical cancer control strategy. The introduction of the HPV vaccine constitutes a shift in health policy and health promotion practice. The aim of this study was to investigate knowledge, beliefs and communication preferences of the Greek public with regards to the HPV vaccine.

**Methods:** Six focus groups (5–8 participants) were conducted with unvaccinated teenage girls, young women and mothers of vaccine-eligible girls, recruited via schools and universities. Pre-focus group questionnaires consisted of: socio-demographic details and a knowledge questionnaire. The discussion guide was based on issues raised by relevant studies such as disease severity and personal risk perception, perceived benefits and barriers to vaccination, etc. Participants were also shown three types of stimuli namely, a leaflet, an expert interview and a documentary containing equivalent information on HPV and the HPV vaccine. Thematic analysis was used for analysis of the qualitative data.

**Findings:** Findings revealed that participants had low to moderate knowledge levels on HPV and cervical cancer. Beliefs specific to the HPV vaccine as well as general beliefs about medicines and their use in everyday life were prevalent. The vaccination dilemma was exacerbated by the conflicting advice received by health professionals coupled with the difficulties participants experienced in evaluating relevant health information. Participants judged all stimuli according to content and format but the documentary was their preferred health information communication option.

**Conclusions:** Findings could contribute to the understanding of health decision making with regards to preventive vaccination and help formulate recommendations for health policy adjustments.

## Background

Every year in Europe 60,000 women develop cervical cancer (CC), the second most frequent cancer in women under 45, leading to 30,000 deaths (Ferlay, Bray, Pisani, & Parkin, 2002). The Human Papilloma Viruses (HPVs) are a family of common viruses implicated in the development of cervical cancer (WHO, 2007). As of January 2008, HPV vaccines against HPV types 16, 18, 6, 11 have been approved for use in a number of countries worldwide. According to the World Health Organisation (WHO) reducing the burden of cc will depend on access to HPV vaccination as well as screening and treatment services (WHO, 2007).

The prevalence of HPV infection in Greek women is similar to other countries in Europe and ranges between 22.7% and 33.1%, which however is reduced to 5.9% when screening for high-risk viruses such as HPV 16 and HPV 18 (Agorastos et al., 2009b; Argyri et al., 2013; Stamataki et al., 2010). However, in Greece HPV vaccination is given free of charge but not supported by an organized effort to increase participation and therefore coverage is estimated at only 9% of the target population (ECCA, 2009). Several Greek studies have reported varying rates depending on the target group under investigation. Tsakiroglou et al. (2011) report an 11% in their sample consisting of women of all ages, Donadiki et al. (2012) report a 25.8% in their sample of higher education students, whereas, finally, Papagiannis et al. (2013) report a much higher 44.3% in their sample of students in the health professions. Furthermore, according to Agorastos et al. (2014) acceptance of anti-HPV vaccination appears to be decreasing over time (from 85–89.9% annually during 2005–2008 to 64.4–60.5% during 2009–2010), a noteworthy occurrence as it coincides with the introduction of the HPV vaccine in Greece in 2008.

Dempsey and Davis (2006) had not only cautioned for careful planning of HPV vaccination implementation, since HPV is the first-ever vaccine against a sexually transmitted disease and could set the tone for what might follow (e.g. an HIV or herpes vaccine), but had also identified potential barriers to HPV vaccination as early as 2006. Since then, a large body of international research investigating these factors has been published. Specifically, women’s lack of knowledge on HPV and the HPV vaccination as well as lack of information about benefits have been highlighted as one of the most important factors negatively affecting the vaccination decision (Donadiki et al., 2014; Mortensen, 2010; Sotiriadis, Dagklis, Siamanta, Chatzigeorgiou, & Agorastos, 2012). These were closely followed by women’s personal beliefs that the vaccine is too new, is unsafe or will cause adverse effects (Haesebaert et al., 2012; Papagiannis et al., 2013; Zimet, Weiss, Rosenthal, Good, & Vichnin, 2010)

A large, U.S. survey study conducted by Zimet et al. (2010) revealed that non-vaccinated women between 19 and 26 years of age listed ‘belief that the vaccine is too new’ (35.4%), ‘not having enough information about the vaccine’ (31.7%) and ‘concerns about side effects’ (24.4%) as reasons for not getting vaccinated. A similar study investigating drivers and barriers among Danish women aged 16–26 conducted by Mortensen (2010) found that lack of information with regards to the benefits of HPV vaccination for older or sexually active women was among the reasons for not going through with the vaccination. A French study conducted by Haesebaert et al. (2012) investigating women’s knowledge and attitudes towards CC prevention discovered that concerns about possible side effects constituted the main barrier to vaccine acceptance. Beliefs such as low perceived benefits from the HPV vaccination were more likely to be reported by unvaccinated individuals in a study by Donadiki et al. (2014).

Furthermore, in a Greek study by Papagiannis et al. (2013) participants also listed ‘fear of vaccine safety’ as the main reason for declining vaccination while they were less likely to vaccinate if they had received information from the mass media compared to other sources (e.g. Hellenic Center for Disease Control and Prevention). Moreover, in a previous study by Sotiriadis et al. (2012), the reason given initially was ‘insufficient knowledge’. However, as the authors interestingly note, this was soon substituted by ‘fear of vaccination adverse events’ which coincided with negative media publicity after the introduction of the HPV vaccine (Sotiriadis et al., 2012).

Healthcare providers (HCPs) have always constituted a formal source of health information for the general public (Logan, 2008) however nowadays the mass media offer much more exposure to health information, successfully placing health issues in everyday conversations or triggering dialogues between healthcare users and providers (Arroyave, 2012). Though the media offer health information in abundance, literature published in the past thirty years has shown it to be at times deceptive, inaccurate, contradictory, alarmist, incomplete, or even outdated (Arroyave, 2012).

In Greece, healthcare providers recognized the HPV vaccine as an important medical innovation, even though had concerns about its safety, effectiveness and long-term impact. Hence they did not consistently recommend vaccination and sometimes advised against it (Karamanidou and Dimopoulos, 2016). These conflicting opinions in conjunction with the inconsistent reports broadcasted by the mass media have certainly contributed to the uncertainty felt by the general public (Agorastos, Chatzigeorgiou, Brotherton, & Garland, 2009a). According to Diffusion of Innovations theory uncertainty about consequences can constitute an important obstacle to the adoption of such an innovation (Rogers, 2003).

The investigation of complex aspects around the HPV vaccination behaviour is particularly salient in Greece where HPV vaccines are provided on demand after prescription from a healthcare professional. Three targeted groups were chosen to participate in the present study: teenage girls (12–17) who are the main target population for HPV vaccination, mothers of vaccine-eligible girls who are likely to influence their children’s decision or make it for them and young women (18–26) who are within the age range for vaccination but more likely to decide this for themselves.

To the best of our knowledge, there have been no other qualitative research studies to date in Greece since the release of the HPV vaccine in 2008 investigating knowledge, beliefs and communication preferences of critical segments of Greek teenage girls, young women and mothers in relation to the HPV vaccine.

Research questions included:

1. What is the level of knowledge of mothers, teenage girls and young women regarding HPV and the HPV vaccine?
2. What beliefs do mothers, teenage girls and young women hold with regards to the HPV vaccine?
3. How do mothers, teenage girls and young women perceive different modes of health information communication regarding HPV and the HPV vaccine and which is judged as the preferred mode?

## Methods

### Design

This was a cross-sectional study employing qualitative methodology. Specifically, qualitative data was gathered via focus group discussions. Some quantitative data was gathered via pre-focus group questionnaires in order to provide a reference frame within which the qualitative data would be analyzed and interpreted.

### Sample

Six focus groups (5–8 participants each) were conducted with teenage girls, mothers of vaccine-eligible girls and young women recruited from two major Greek cities; Athens and Thessaloniki. Participants responded to invitations funneled (bullet-in boards, mailing lists) through key institutions namely local schools and universities.

Two focus group discussions were held with each of the following: (1) unvaccinated teenage girls between the ages of 12 and 17; (2) unvaccinated young women between the ages of 18 and 26; (3) mothers of unvaccinated girls between the ages of 12 and 17.

### Materials

#### Questionnaires

Pre-focus group questionnaires consisted of questions on socio-demographic data and a knowledge scale. Socio-demographic questions included age, education, personal history of cervical cancer, HPV awareness and previous vaccine recommendation by a healthcare professional.

The knowledge item scale (Gerend & Shepherd, 2011) included 10 true or false items concerning certain HPV facts, cervical cancer and cervical cancer screening facts, etc. One phrase was modified from ‘HPV is the least common sexually transmitted infection in the U.S.’ to ‘HPV is the least common sexually transmitted infection in the Western world’. Responses were coded so that each correct answer received a score of one, with ‘incorrect’ or ‘unsure’ answers coded as zero. The knowledge summary score resulted from the sum of correct answers with higher scores reflecting higher levels of knowledge.

#### Discussion guide

The discussion guide was developed drawing on the existing relevant literature. Issues put forward in the study by Kang and Kim (2011) as well as Flynn and Ogden (2004) provided useful insights about the topics worth covering. These topics included: perception of personal risk, beliefs regarding the severity of HPV infection and cervical cancer, beliefs about vaccines in general and the HPV vaccine in particular, perceived benefits and barriers, vaccine efficacy and safety, recommendation or influence of significant others, behavioral intentions and experiences of issues surrounding HPV, reliability and quality of information received by the media, etc.

#### Stimuli

Participants were asked for their thoughts primarily with regards to the content and format of the stimuli. Comments were also encouraged with regards to the credibility of actors and information, but also presentation and language. The following stimuli were shown:

##### Documentary

‘Catching Cancer’. This documentary presented a new outlook on the way viruses play a role in some cancers specifically, the role of HPV in CC and the introduction of the HPV vaccine. Permission to use this documentary was granted by the Australian Broadcasting Corporation and a subtitled version was provided by the CAID center which had held a screening during the International Science Film Festival in Athens, Greece. Participants were shown minutes 12.00 to 22.10. The documentary is available online: http://www.youtube.com/watch?v = XWJXqoaQU-k

At first, a couple is shown dancing and the term ‘cancer a deux’ is explained as the simultaneous occurrence of cancer in both partners. The narrator continues to explain that the cause of cancer was hypothesized as early as 150 years ago after consistent observations of no incidence in individuals with no sexual activity such as nuns and in contrast higher incidence in individuals with multiple partners. The next section shows an interview with Professor Zur Hausen, a Nobel Laureate, who introduced the radical idea that HPV, a virus that causes genital warts, also causes cervical cancer in the 1970s. The narrator goes on to explain the scientific methods by which this hypothesis was tested. After a decade of research Zur Hausen’s group succeeded in proving that two types 16, 18 were present in cervical cancer tumors and thus labeled them as carcinogens capable of turning healthy cells into cancer cells. Next, Vanuatu is shown in the background, as the personal story of a woman with cervical cancer is told. Subsequently, Ian Fraser and his Australian team are introduced as they arrive to the island and administer the HPV vaccine to teenage Vanuatu girls. He explains that the HPV vaccine, a vaccine specifically designed to prevent cervical cancer, has been the result of 15 years of research and a very useful add-on to screening and other ways of controlling cancer available in the western world.

##### Expert discussion

A discussion with a male Greek gynecologist and HPV expert who had been invited to discuss HPV and the HPV vaccine with two female journalists/ presenters within the context of a health program broadcasted by a Greek state channel. Participants were shown minutes 00.00 to 9.32. The discussion is available online: http://www.youtube.com/watch?v = kgYm_u-rvpQ After the expert is introduced, he is asked about the HPV virus and its link to cancer, the HPV vaccine, vaccine safety, efficacy, vaccination target groups, HPV vaccination and smear test as cervical cancer prevention strategies, HPV infection treatment, HPV infection and its link with sexual behavior.

##### Leaflet

The Greek Organization of Child and Pediatric Gynecology (www.aretaieio-obgyn.com) produced a leaflet titled ‘Get vaccinated today! Vaccination to prevent human papillomaviruses causing cervical cancer and other serious diseases’ and followed a question and answer format. It presented the main facts about HPV and the link to cancer, HPV virus transmission, treatment of the HPV infection, the HPV vaccine, including safety, efficacy, mode of delivery, and vaccination target age groups. The leaflet could be found in Obstetrics and Gynecology clinics.

### Procedure

The focus groups discussions were conducted at the end of 2014. An invitation to participate was forwarded to key institutions with an aim to attract interested individuals who wished to participate in the study. A screening paragraph was developed to help recruitment in a standardized way. The focus groups were structured in the following way: administration of the pre-focus group questionnaire, discussion based on the discussion guide, presentation of all three stimuli followed by a specific discussion on these stimuli. At the beginning of each session, the nature and aim of the study were introduced and the structure of the session was explained. In order to control for order effects, the order of stimuli presentation alternated. For example, in the first focus group discussion the order with which the stimuli were presented was 1, 2, 3 for the second focus group it was 2, 3, 1, while for the third, it was 3, 1, 2, etc. The focus groups took place in schools and universities in Athens and Thessaloniki and were video recorded.

### Analysis

The qualitative data were analyzed by employing thematic qualitative analysis (Braun & Clarke, 2006). Following the identification, revision and analysis of themes, appropriate extracts were selected from the focus group discussions in an effort to ensure that similarities and differences across accounts are shown and different perspectives are represented (Noble & Smith, 2015). The analysis was performed by both researchers separately (inter-rater reliability score: 98%). The translations of all quotes from Greek into English were firstly performed by the principal investigator and subsequently by an applied linguist. Disagreements on terminology and choice of wording were negotiated and resolved. This was done in agreement with cross-cultural researchers suggesting that the translation process should be conducted by two separate translators (Esposito, 2001) and that researchers should stay in the original language as long as possible (Van Nes, Abma, Jonsson, & Deeg, 2010). Following these recommendations enhances the validity of cross-English qualitative research by reducing the loss of meaning since thinking and language are so closely related. The researchers who performed the analysis are Greek, hold Ph.D. degrees, were educated in the UK and have experience in conducting qualitative research. All of these factors can positively affect the quality of translation (Birbili, 2000) and subsequently research findings as well (Noble & Smith, 2015). In closing, it should be mentioned that the researchers are of a different gender and age group. This fact aided reflexivity throughout the course of the study as it created a dialogue which led to the contribution of complementary perspectives.

### Ethics Statement

During recruitment participants were informed about the purpose of the study, the organization hosting the research study, the study’s procedure, their right to anonymity and confidentiality, their right to get informed about the findings after the end of the study and finally their right to withdraw from the study at any point without giving a reason. All participants gave their consent for participation and video recording of the discussion. Teenage girls’ recruitment was conducted via the schools after a consent form was obtained, signed by their parents. For confidentiality purposes, an abbreviated version of the group type (MG, TG, YG) and sequence (1, 2) as well as a participant number (N1, N2 … ) accompanies every quote. This study adhered to the current Research Ethics guidelines as articulated in the European Commission’s ‘Ethics for Researchers’ (7th Framework) and the declaration of Helsinki.

## Findings

### Quantitative data

#### Demographics

The sample included 13 mothers out of which 2 were high school graduates and 11 were higher education graduates. Also, 11 university students and finally 16 school students out of which 8 attended Gymnasium (low secondary school) and 8 attended Lyceum (upper secondary school). The vaccine was recommended by a health professional to 60% of the sample. A small portion of the sample (5%) had been informed by a health professional that they had precancerous cervix lesions.

#### Knowledge

The mean HPV knowledge sample score was relatively low with a mean score 3.8 (SD = 1.94) out of a possible maximum score of 10, while mean knowledge scale scores differed across the three age groups (Table 1).

**Table 1.** Age and knowledge scores across the three groups.

	Mothers	Young women	Teenage girls	Group total
Age range	40–56	18–21	14–16	14–56
Mean age	47.8	19.1	15.4	26.4
Knowledge score range	0–6	2–8	2–5	0–8
Mean knowledge score	2.6	5.4	3.6	3.8

**Figure 1.**
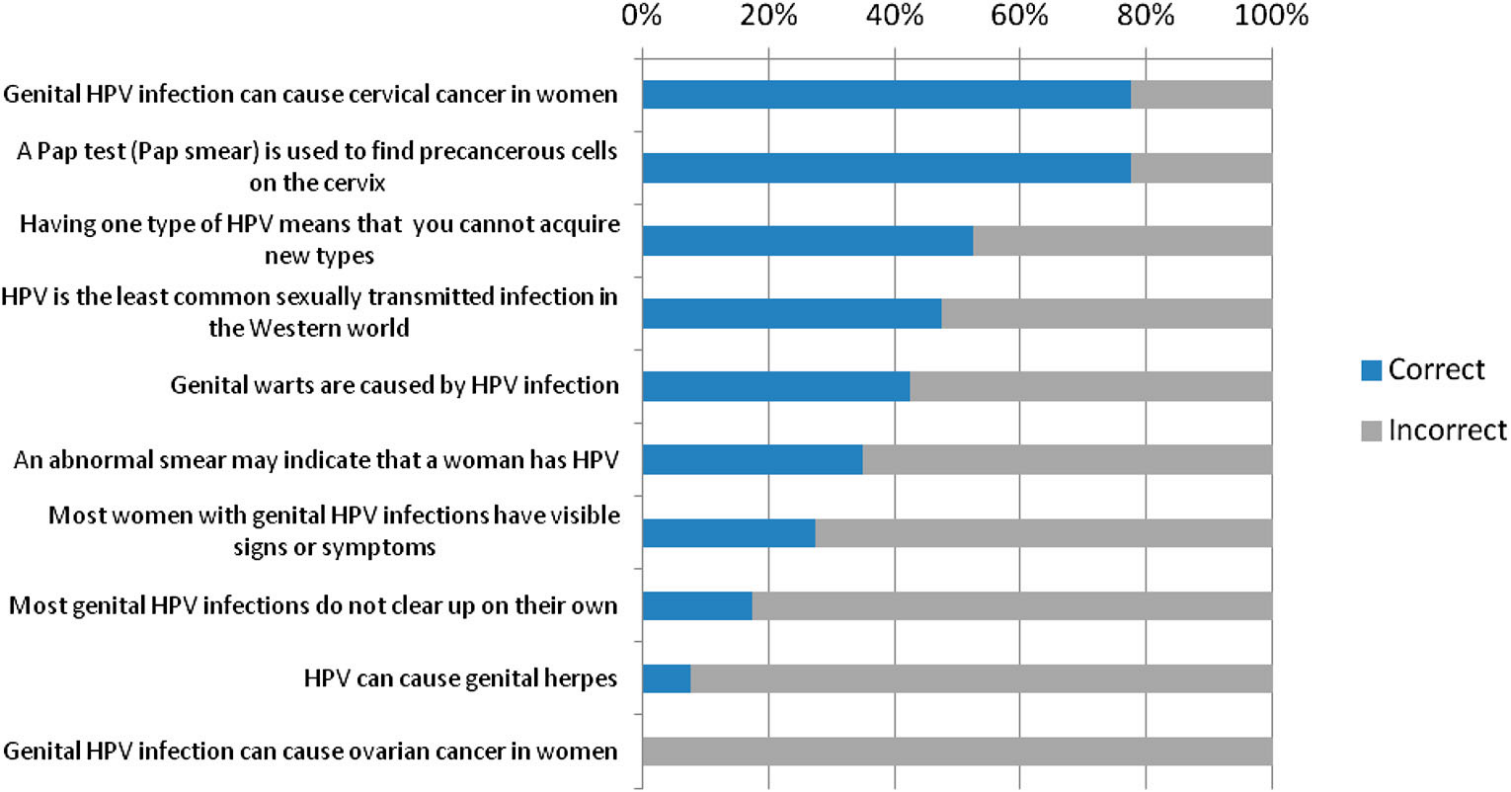
Percentage of correct and incorrect responses per knowledge item.

Mann–Whitney *U-*test analyses were performed and revealed significant differences between mothers and young women (*U*= 26,000, *p* = .007) as well as between young women and teenage girls (*U* = 36,000, *p* = .008). The percentage of correct and incorrect responses for each knowledge item is shown in Figure 1.

## Qualitative data

The themes are individually analyzed and presented along with supporting representative themes (Table 2).

**Table 2.** Qualitative analysis themes.

Theme	Subtheme	Representative quote
The HPV – cervical cancer link *This theme focuses on participants’ perceptions of HPV as a virus that is responsible for changes at a cellular level causing CC. Also, their perceptions around the efficacy of CC prevention and the severity of CC as a disease.*	Perception of CC cause	‘*so cc begins with a virus? It is an infection*? (MG1, N1) ‘*things mutating and cancer appearing … this is something we have heard in other cancer cases i.e. a cyst that can mutate*’ (MG1, N5) ‘*an HPV infection can turn into cc cancer in the future*’ (YG2, N4)
	Perception of CC severity	‘*It is an illness that you can cure if you find it early, there are methods, but these illnesses do not cause symptoms at first, only at a later stage*’ (TG2, N3) ‘*a serious health problem but a treatable one if caught early*’ (MG1, N1)
The HPV vaccine *This theme focuses on participants’ beliefs about the efficacy, necessity and innovation of the HPV vaccine. Also, their concerns and perceived barriers to vaccination.*	Necessity	‘*It is the only vaccine against cancer … *’ (MG1, N6) ‘T*o know there is such a tool and not use it … ?’* (MG2, N5)
	Concerns	‘*we don’t know if it’s safe … because women who have had it (the vaccine) have not had children yet … in order to see if there is a consequence’* (TG1, N8) ‘*Why would I have it and put my life in danger?*’ (YG2, N4)
Distrust *This theme focuses on participants’ distrust towards the pharmaceutical industry, the Greek state and medical science. It discusses motives, state responsibilities and epistemological beliefs.*	Pharma industry	*‘we are distrustful because at our age we believe that there is a financial motive behind everything’ (MG1, N1) ‘Every year my husband had the flu vaccine because he has heart problems, this year the same doctor said don’t bother with it … it makes you suspect things … I was just taken by surprise’* (MG1, N6) ‘*Do they test it on people in Africa?’ (MG1, N2)*
	State	‘*Greece is ambivalent with regards to this in contrast with countries abroad where there is a clear cut recommendation even if it’s based on cost – in Greece we improvise as always’* (MG2, N4)
	Medical Science	‘*contraceptives, IVFs, we reached very high levels of pregnancies … yes yes it is the way to motherhood etc however, the consequences are just now starting to show … the women are getting older, the children are getting older and all the problems are just starting to show’* (MG1, N3) ‘*These are vaccines … who knows what they are going to say after 10 years about them … ’* (MG2, N1)
Beliefs about medicines and their use *This theme focuses on participants’ beliefs about medicines. It discusses their very nature and their frequency of use in every aspect of life.*	Over-medicalization of life	*‘Basically now, it has been become really dangerous to come in contact with a man in this way because everything might pose a problem, the condom, the vaccine … get it or don’t get it … I at least am worried*’ (TG2, N3) ‘Ι*t is a bit excessive … have the vaccine, use a condom … .’* (TG2, N6)
	Overuse of medicines	‘*How many vaccines do we have to get in order to protect ourselves*?’ (MG1, N2) ‘*Oh unsold vaccines, for swine flu, the bird flu … run*!’ (MG1, N4)
	Medicines are poison	‘*All vaccines are taint’* (MG2, N1) ‘*vaccines are part of everyday life but one still makes the sign of the cross beforehand’* (MG1, N2)
The vaccine dilemma *This theme focuses on the dilemma all participants faced. It discusses the role of health professionals namely informing, recommending and influencing the decision. Also, it discusses the decision making process and specifically the actors involved, the health literacy needed to evaluate information and the resulting emotional consequences.*	Actors, health literacy and emotional consequences	‘*Whichever way you look at it you are taking a risk whether you go ahead with it or you don’t*’ (MG2, N2) ‘*Even if we go through with it, at the back of my mind there will still be this fear … *’ (MG2, N1) ‘*you have a dilemma – do it, not do it and imagine the guilt if it appears’* (MG1, N5)
	Health care professionals’ role	‘*The doctor said that he can take care of women with routine check-ups and won’t let anything develop into cancer. Also, that having the vaccine increases your chances of getting multiple sclerosis*’ (TG2, N3) ‘*However, opinions differ … . I have not decided for my daughters yet because my gynecologist said ‘What you haven’t done it yet? My sister though, who is a doctor, told me to wait … there are trials being conducted etc. and this is a cause for concern*’ (MG1, N6) ‘*I know of a case where the vaccine was administered after the infection and the reproduction of the virus was stopped’* (YG2, N4) ‘*The doctor said I should go for the one against the two types vs. the one against the four types as the latter has many side effects she did not want to tell me about so I don’t get alarmed’* (MG1, N4)
HPV and sexuality *This theme focuses on the link between HPV and sexual activity. Specifically, it discusses the symbolic value of the HPV vaccine as permission for the sexual debut. Also, it discusses participants’ high behaviors with regards to sexual activity.*	Perceptions of HPV risk	‘*Individuals aged between 17- 30 because they are searching for their sexual identity’ (YG2, N3) ‘Women with an active sexual life get it’* (TG1, N6) ‘*all of the sexually active population from 15 to 95’* (MG2, N8) ‘*warts make their appearance when there are multiple sexual partners’* (MG1, N2)
	The vaccine as a ‘coming of age’ milestone	‘*She has to be informed about what it is that she is about to do … and what dangers she is exposing herself to by commencing a sex life. She needs to be more mature and this is a milestone following which you should conduct yourself more responsibly as if you are an adult although you are not one yet’* (MG2, N5)
Needs *This theme focuses on participants’ self-reported needs for appropriately communicated health information and sexual health education.*	Appropriately communicated health information	‘*Need we say this out loud? It is a matter of management! If it played on TV all day and the advice was to go ahead with it, do it, … . at some point … it is your kid, what would you say, no I won’t do it !?’* (MG1, N2)
	Sexual health education	*‘It will be different for them to hear this information coming from a doctor as opposed to coming from their mum’* (MG1, N4) ‘*these are children who can think they are invulnerable to a million things after getting vaccinated and then the opposite result will be achieved in that they won’t be protected against everything else’* (MG2, N4)

### The HPV-cervical cancer link

#### Perception of CC cause

The majority of participants were aware of the link between HPV and cervical cancer (cc). Specifically, that CC is a possible but not definite outcome of the HPV infection.

The idea that a virus may cause cancer was surprising to a few participants in one of the mothers’ groups who had no prior knowledge of the HPV-CC link. After further discussion within the group this concept did not seem as outlandish as initially thought by participants who managed to think of other examples of benign or non-life threatening conditions evolving to cancer i.e. colon polyps to colon cancer.

#### Perception of CC severity

Cervical cancer was judged as a serious health issue which could have consequences on a woman’s reproductive ability. Moreover, participants were acutely aware of the importance of symptoms with regards to seeking medical help and benefits of early detection of CC. Also, they realized current medical advancement allows for the successful treatment of CC. Finally, they recognized the smear test and the HPV vaccine are both effective ways of CC prevention.

Overall, young women and teenage girls were more knowledgeable than mothers on HPV and CC issues, a fact also observed from the quantitative data. However, all participants stated that they knew much more about other forms of cancer (i.e. breast cancer) compared to CC and much more about other sexually transmitted infections (i.e. HIV) compared to HPV. This is not surprising as these health issues either have received much more publicity and/or have been at the center of the public health arena for a longer period of time.

### The HPV vaccine

#### Necessity

Necessity beliefs were expressed by participants revolving around the idea that the vaccine is an important medical innovation, a powerful tool for all to use and the only vaccine that can protect against cancer. Great focus was given on the regret they would feel if they did not get vaccinated and during the course of their lives contracted an HPV infection. Furthermore, participants were aware that HPV vaccine can protect against the most frequently seen, high-risk strains of HPV and thought it was an effective means of CC prevention.

As these were unvaccinated individuals, in their attempt to make a decision, they weighed their concerns about the risk of getting vaccinated against the benefits of protecting oneself from cancer. This negotiation involved an active search of information about the HPV types the vaccine could guard against and the side effects commonly experienced by vaccine users.

#### Concerns

All of the participants cited the novelty of the vaccine as the main barrier to vaccination. Specific concerns mainly included possible long-term consequences and short-term side effects. Other concerns expressed included the possibility that individuals have an allergy to an ingredient within the vaccine or have a weak immune system and do not respond to the vaccine in the desired way. Finally, the possibility that the virus manifests itself after it enters the body through the vaccine as if the person has been infected.

The information communicated by health care professionals naturally influenced their beliefs about the HPV vaccine and their attitudes towards vaccination. Participants stated that they had received conflicting advice from healthcare professionals in terms of the vaccination recommendation, a fact which made them question the reliability of the vaccine itself. Their concerns were further reinforced by testimonials they had read or heard about, of individuals receiving the HPV vaccine and suffering serious side effects such as paralysis.

### Distrust

#### Pharma industry

The majority of participants voiced the opinion that healthcare professionals are eager to promote the administration of HPV vaccine and other medicines because they have an ulterior motive. Specifically, health care professionals gain financially from pharmaceutical companies and therefore medical decision making is essentially shaped by their product promotion than by strictly medical purposes. They also voiced concerns about the procedures pharmaceuticals followed to get a product from research to the market, the location and participants of clinical trials and wondered about the ethical issues that might arise and how these are being handled.

#### State

Doubts were expressed solely in the mothers’ group about the way the state is recording and categorizing incidents of HPV vaccine side effects. Furthermore, participants had reservations about whether the appropriate state agency is feeding back this information to the pharmaceutical companies or to the health care professionals in a systematic manner. Most importantly, they had a desire to know the manner in which members of the public can report incidents related to the vaccine. This was especially salient due to the fact that the vaccine is not administered in the context of state vaccination effort but rather can be administered individually at a local pharmacy store without a follow-up. Finally, mothers wondered whether this information was ever made public through formal sources.

#### Medical Science

This theme pertained to the advance of medical science, a continuous process during which the old becomes outdated and gives way to the new. There was a consensus among participants that a lot of medical recommendations once considered imperative were disputed sometime in the future. Older participants mentioned as an example the polio vaccination, which was converted into an injectable form and consequently lost its potency. Medical science itself is questioning medical innovations after consequences start to show. Participants’ indecisiveness with regards to the vaccination was enhanced by their concern that this medical innovation, namely the HPV vaccine, might not be the medical recommendation a few years from now.

### Beliefs about medicines and their use

#### Over-medicalization of life

Another issue raised by participants was what they refer to as ‘over-medicalization of life’. Participants of all groups felt that every simple act of life in the present day and age has to be a cause for concern. Mothers regretted the fact that their daughters cannot enjoy the freedom and carelessness they themselves had enjoyed in their youth. On the other hand, young women and teenage girls expressed the notion that the complexity of this matter which should be simple and driven by emotion has instead become a series of calculated actions and a cause for concern and anxiety which definitely kills the magic of love affairs.

#### Overuse of medicines

Another issue raised by participants was the overuse of medicines. Participants thought that there is too much medication on offer and it cannot all be necessary. Participants were also quite critical about the urgency under which these vaccines are offered implying overuse by the medical community. Some participants provided counterarguments such as that medicine is evolving and life expectancy in the western world has been prolonged specifically due to the use of medication. However, others remained apprehensive about the use of medicines despite their widespread presence in everyday life.

#### Medicines are poison

A small number of patients from the mothers and young women’s groups voiced the opinion that all medicines are addictive or harmful as they contain chemicals bound to be dangerous for the human body. They made a point of stating that they looked for a herbal or natural alternative to most medicines. With regards to vaccinations in particular, a minority of participants thought that they debilitate the human organism and prevent it from fighting independently. Finally, a minority of mothers expressed the wish to talk to naturopaths or homeopathic doctors about HPV vaccination. Specifically, listen to a debate of an expert ‘outside the system’ against a traditional western medicine doctor.

### The vaccine dilemma

#### Actors, health literacy and emotional consequences

Young women had made their own decision not to get vaccinated. Teenage participants reported that health decisions were always taken by the family upon the recommendation of a healthcare professional. In contrast, some mothers thought that their teenage daughters should be involved in the decision of the HPV vaccination. Others thought that under the law the decision should rest with them since their daughters did not have the knowledge or criteria on which to base a decision. During this part of the discussion, mothers displayed an array of negative emotions resulting from the cognitive process of decision making. Concern, fear and guilt were the most prominent emotions.

The decision-making process was based on participants’ evaluation of risk. It is noteworthy that participants tried to make sense of the statistical figures they heard during the documentary and expert discussion. Some participants became confused when they tried to combine the information they heard from both stimuli. For example, in the documentary, it was mentioned that 500,000 women die of CC every year. And in the expert discussion, it was mentioned that 80% will have the virus but only 2% will actually get cancer. Also, the level of protection a condom can offer to women is approximately 70%. Some participants thought that since only 2% of women out of 80% will get cancer and if one considers the protection of condom use and cervical cancer screening, it is not really necessary to get the vaccine which does not even provide 100% protection from all HPV strains. Others thought this level of protection was satisfactory. It is noteworthy that the decision making concerning the vaccine was judged on facts the medical significance of which could not really be judged by participants, making an informed decision quite a challenge.

#### The role of HCPs

Participants shared their stories with regards to advice they had received from health professionals. The majority of participants reported negative, conflicting or complex advice. Sometimes, mothers were told to wait until their daughters got a regular cycle. In other cases, participants had received a recommendation but only for one of the two vaccines. Other participants were told that the decision should be made by them because the vaccine is still very new and no conclusion with regards to its effectiveness can be drawn yet. Even when the advice was not conflicting, namely both the pediatrician’s and the gynecologist’s advice coincided, participants still could not come to a decision or delayed making the decision due to their own concerns. Conflicting advice led to second or third opinions that further complicated the decision-making process.

### HPV vaccine and sexuality

#### Perceptions of HPV risk

Some participants evaluated HPV as a normal risk that all individuals take by embarking on a sex life however some stereotypes were also voiced. Specifically, some teenage participants thought that really promiscuous behavior puts individuals at risk while some young women thought that individuals who did not take precautions such as use a condom were most at risk.

#### The vaccine as a ‘coming of age milestone’

This theme only came up in the mothers’ groups. Mothers in both groups were distinctly aware that sexual activity could expose their daughters to dangers such as the HPV virus. However, the majority of participants were undecided about the HPV vaccination. Still they stated that they used the HPV vaccine as leverage in discussion with their daughters to delay their sexual debut as much as possible. When participants came to realize that this was a common strategy used by many, they found it particularly entertaining. Using self-sarcasm, e.g. ‘we are awesome’ they admitted this was an agonizing attempt to protect their daughters from what is essentially an integral part of growing up. In their mind, the vaccine constituted a coming of age milestone; hence they tried to delay this decision as much as possible.

### Needs

#### Appropriately communicated health information

Teenage participants stated that their source of information was mainly their family, friends, doctors and peer and that if they were to look for more it would be on the internet. Young women had heard about HPV at school or through their studies while others recalled a ministry of health campaign for the vaccine. Mothers had heard about HPV from the media or read about it on the Internet, while some had read leaflets in the health service. They had asked their own doctors (i.e. gynecologists), or their daughters’ doctors but also asked for the advice of relatives who happened to be health professionals of any kind.

All participants stressed that they had information gaps around HPV and HPV vaccination. Among the issues participants from all groups would like to receive information on are: symptoms of HPV infection in women and men, pregnancy and HPV, the difference between the first dose of the HPV vaccine and subsequent booster doses, the possibility of administration after sexual debut, the possibility of a causal link between prostate cancer and HPV, ways of transmission, detection and cure, the vaccine’s active ingredient, the length of time needed for an infection to resolve itself, the possibility of being more prone to other strains of the virus if already infected by one strain, the role of men in transmission, the number of sexual partners that puts one at risk, HPV vaccination for boys, vaccination recommendations in other countries, precise recommended age of vaccination and the exact way the vaccine works against the virus.

When asked what would sway their opinion in favor of the HPV vaccine participants highlighted that the media is a powerful source of information for the public concerning public health matters. However, they mentioned that the specific health issue as portrayed in the media rests on the motivation of fear over certainty that vaccination is the right decision.

#### Sexual health education

Mothers thought the HPV vaccination should be given by experts such as physicians in the context of a sexual health talk. Participants admitted the limitations they had in their roles as mothers due to the fact that they had no expert knowledge or authority in their daughters’ eyes to deliver such sensitive information.

Young women and teenage girls thought recommendations should come from doctors but also from experts such as pharmacists. They believed that information about sexual health should be given at the end of primary school as well as junior high and during high school.

Participants of all groups highlighted the danger of administering the vaccine outside the context of a sexual health talk. Furthermore, that it should be made clear that the vaccine cannot provide complete safety from all STDs, so that vaccinated individuals are not encouraged to engage in the risky sexual behavior.

## Judgments on the stimuli

### General

None of the participants had ever seen any of the stimuli so their reactions were spontaneous. Participants read and watched the stimuli attentively. They noticed inconsistencies, which reveals that they were very interested in the topic and did not receive the information passively. A summary of participants’ judgments on the stimuli format and content are shown in Table 3. Participants appreciated each format for different reasons. Remarkably, they were won over by the documentary which provided a context to the HPV vaccine and was more impressive in its presentation. In terms of content, they would prefer a balanced approach to the HPV dilemma, which is more compatible with their own experiences and does not reinforce their concerns but rather provides answers to the issues they are struggling with, i.e. side effects.

**Table 3.** Participants’ judgments on the three stimuli.

	Leaflet		Expert interview		Documentary	
	Positive	Negative	Positive	Negative	Positive	Negative
Format	Definite structure Helpful Can go over the information more than once	Unattractive cover Cryptic title Does not have a clear aim	Resembles reality Live interaction raises interest Spontaneous discussion allows for topics to come up	Presentation of the doctor as an ‘expert authority’	Impressive Combines visual and auditory stimuli Easier to follow Provides a storyline, context and background	N/A
Content	Satisfactory level of information	The issue of men is suddenly introduced in the last section Does not address the issue of side effects	Very Informative	Not convincing Too adamant/ certain No information on statistics or vaccination sites Does not address the issue of side effects	Convincing Offers specific/ detailed information ‘Personal meeting’ with the researchers who developed the vaccine Researcher presents the vaccine as one of the weapons against CC	Negative connotations from the presentation of the mass vaccinations Does not address the issue of side effects

### Teenage participants

Teenage participants thought the level of information was appropriate and the content of the stimuli was equivalent. Judgments with regards to the expert interview revealed that teenage participants felt that the doctor was too confident in the way he was delivering the information. This came in sharp contrast with their own experience. Participants seemed to be a bit puzzled when they thought back to the contradictory information they had received. One participant said ‘I was surprised that he talked with such confidence  …  how is it possible for some to be so certain and others to … say something different’ (TG1, N2). The fact that the doctor seemed so adamant, concerned them. Another participant said ‘He is so certain (about the vaccine), that it overpowers everything else’ (TG2, N3). Participants felt that the leaflet was not very attractive and its title did not give away the topic. Also, they thought that it needed a better cover and a clearer aim. The documentary received only positive judgments i.e. this information is easier to follow in a documentary format due to its combined use of image and sound. When asked about other criteria such as format and structure, some participants thought the leaflet was more helpful as it had a definite structure and one could go over the information more than once. In contrast, some thought the discussion with the expert resembled reality more since the interaction between the journalists and the expert offered more information than the leaflet as the discussion was spontaneous and other topics could come up. However, the documentary was the preferred way of communication out of the three stimuli presented. The reasons given by participants were that they were able to put the HPV vaccine since they had a chance to ‘meet’ the researchers who developed it.

### Mothers

Participants in the mothers’ groups said that the extract with the expert was informative. Moreover, they were surprised about some of the facts communicated such as that HPV can be transmitted even without full sexual intercourse or that 50% of youngsters have started to experiment sexually at the age of 14. They commented on the fact that the expert was certain and ‘he thought it natural that it (the vaccine) had to be done’ (MG1, N6). Another criticism was that there was no indication of neither the time the vaccinations took place initially nor the statistical data available for Greece or Europe. The fact that the doctor appeared as an expert authority was not seen positively by some participants. The leaflet was judged as containing a satisfactory level of information. One criticism was that the issue of side effects is not touched upon. On the contrary, the issue of men is suddenly introduced in the last quite irrelevantly of any previous information. Overall, the documentary was judged positively. One aspect that caused negative criticism was the mass vaccination that it showed in Africa ‘I was scared by this mass vaccination. A little bit like guinea pigs’ (MG1, N5). The documentary was judged as more convincing and impressive. However, still participants’ reservations concerning side effects were not addressed.

### Young women

Young women’s overall judgments of the stimuli were positive. With regards to the expert discussion, young women found a few inconsistencies i.e. that initially it was mentioned that the vaccine protects against HPV but later it was mentioned that this protection is not at 100%. Also, the expert was judged as not very convincing as it felt like ‘product promotion’ (YG1, N4). Participants thought the documentary was more specific mentioning more information such as booster doses, etc. Moreover, participants pointed out that although the researcher who discovered the vaccine, did not ‘sell’ it as a unique truth or idealize it. Participants were quick to combine information and identify discrepancies in the information offered via the three stimuli. Specifically, the expert expressed the opinion that if a young girl gets vaccinated and gets smears then she will never get cancer. However, in the documentary, it was pointed out that other factors might be at play in the development of CC.

## Discussion

The purpose of this study was to investigate the knowledge, beliefs and communication preferences of unvaccinated Greek adolescent girls, young women, and mothers of vaccine-eligible girls with regards to the HPV vaccine.

This sample of non-vaccinated young women, teenagers and mothers whose daughters had not been vaccinated, had a low to moderate level of knowledge around issues relating to HPV and cervical cancer. Additionally, findings revealed a differentiation among the three groups. Specifically, young women had the highest knowledge score, followed by teenage girls, while mothers displayed the lowest level of knowledge. Nevertheless, all participants showed awareness that they lacked health literacy to assess health information on HPV and the HPV vaccine. The vaccination dilemma they experienced was further enhanced by the conflicting advice received by health care professionals. Indeed, participants realized the severity of cervical cancer, and they perceived the HPV vaccine as a powerful and essential weapon in the battle against it. However, they considered it to be a recent medical innovation and their concerns only fueled their fears concerning vaccination. Furthermore, in a conservative society such as the Greek one, the decision for vaccination was not seen as just a health-promoting decision but inadvertently symbolized young women’s sexual debut. Finally, participants expressed the belief that life nowadays is becoming increasingly over-medicalised due to the overpowering presence of medicine, which turns every simple act of life into something quite complex. Great distrust was shown towards the pharmaceutical industry as well as medicine in general, revealing a range of underpinning negative epistemological beliefs.

Previous Greek studies (Jelastopulu et al., 2016; Notara, Soultatou, & Tselika, 2012) have also identified knowledge deficits and misconceptions in topics such as virus transmission. However, the present study revealed that participants also had specific beliefs concerning not only the vaccine itself but medicines in general. Beliefs about medicines have been shown to affect health behavior decisions in a range of other health issues (Horne et al., 2013; Horne, Weinman, & Hankins, 1999). Bekker, Gough, and Williams (2003) suggested that individuals’ flu vaccination behavior can be influenced by their beliefs about vaccination and not merely by their level of knowledge.

Previous HPV studies have found concerns about vaccine safety to prevent vaccination uptake. Doubts about efficacy due to the newness of the vaccine can also constitute a major barrier to vaccination in adolescents (Bartlett & Peterson, 2011) and parents alike (Gowda, Schaffer, Dombkowski, & Dempsey, 2012). Similarly to the present study’s findings, parents either stated that they did not have enough information to make a decision or expressed concerns that the information provided by health care professionals was biased. Specifically, that vaccination benefits were stressed while side effects were not mentioned.

In the current study, all three age groups held strong general beliefs about the overuse of medications, previously also observed by Koster, Heerdink, de Vries, and Boyvy (2014). This finding is not surprising in light of frequent public announcements of doctors’ overprescribing instances in Greece, until recently the first European Country in pharmaceutical expenditure per capita (Canadian Medical Association, 2011). Participants’ accounts revealed feelings of distrust towards the pharmaceutical industry and its products. Coupled with this, a profound lack of faith in the state was also expressed. Participants even doubted healthcare professionals’ motivation behind the prescription of the vaccines thus betraying a deep-seated belief that there is an underlying secondary gain.

Present findings also suggested that the public is expected to make an important health decision while receiving conflicting advice from the environment. Participants were of the opinion that the HPV vaccine is not a children’s vaccination where no prerequisites, conditions, or patient history are needed. HPV vaccination requires active participation from the recipients at all stages i.e. making the decision, adhering to catch-up doses and even reporting adverse effects. It is noteworthy that none of this study’s participants knew the procedure on how to report an adverse effect following immunization. Furthermore, since there is no formal follow-up in place, participants judged the monitoring system efficacy in a negative manner (e.g. that a hospitalization incident would be recorded and attributed to the specific vaccination).

Furthermore, participants stated that they had received conflicting advice from healthcare professionals with regards to the HPV vaccine. According to Mårdby, Åkerlind, and Hedenrud (2009) health care professionals can hold different beliefs about medicines. Since health care professionals have a role in communicating information about medicines, their own beliefs could influence the patient consultation and thus have a direct impact on individuals’ health behaviors. Indeed, the strength of the physician’s recommendation has been proven to significantly influence women’s decision to get vaccinated against HPV (Rosenthal et al., 2011). However, studies suggest that health care professionals do not always proceed with an HPV vaccination recommendation due to their own concerns about safety and efficacy (Karamanidou and Dimopoulos 2016). Other factors such as lack of information about the vaccine, time required to discuss vaccination with patients/parents, discussion on sexuality, etc. may also constitute barriers for vaccination recommendation by health care professionals resulting in missed clinical opportunities (Vadaparampil et al., 2011).

The notion that different segments of the public might have difficulty understanding health statistics has been put forward by researchers a number of years earlier. According to Gigerenzer, Gaismaier, Kurz-Milcke, Schawartz, and Woloshin (2008) health risk communication, be it via the web, leaflets or pharmaceutical pamphlets, is often presented in a way that the public cannot comprehend. This has grave consequences in that the public cannot really make informed decisions but it rather is manipulated by emotions brought on by their attempt to understand the received information. This was also evident in the present study from the participants’ attempts to comprehend medical facts included in the shown stimuli. Difficulties in information processing were more likely to reinforce the vaccine dilemma giving rise to a range of negative emotions in participants such as anxiety, guilt, fear, ambivalence, etc. Participants noticed information inconsistencies among the presented stimuli (i.e. the percentage of HPV infections developing into cancer), a fact which further amplified their negative emotions.

Studies on consumers’ perceptions of health information in local media have revealed that participants think that news stories rely on sensationalism and emphatic headlines in order to attract attention and compete amongst themselves (Van Slooten, Friedman, & Tanner, 2013). As evidenced by the findings of Jensen, Scherr, Brown, Jones, and Christy (2013) adult perceptions of cancer survival rankings (i.e. breast, stomach and pancreatic cancer), are distorted and this has been attributed to their misrepresentation in the news. Fewer than half of US newspaper articles about the HPV vaccine contained detailed health information even after the vaccine’s approval by the appropriate authorities (Quintero Johnson, Sionean, & Scott, 2011). The role of the media is key with regards to the delivery of health information which could help to promote positive health behaviors and eventually influence public health. Exposure to news coverage that does not contain an adequate level of information about the HPV and the HPV vaccine, can leave audiences poorly informed and thus lead to ill-informed decisions (Quintero Johnson et al., 2011).

In accordance to Bartlett and Peterson (2011), the present findings suggest that there is a need for current and accurate information on the HPV vaccine. Findings of a recent Australian study suggest that exposure to a detailed informational message as opposed to a brief one increases illness coherence, a psychological construct found to predict HPV vaccination uptake. Illness coherence is not just about learning the facts about a health issue but rather feeling confident about the knowledge acquired and subsequently acting upon it (Sherman, Kilby, Moore, & Shaw, 2017). Moreover, Krawczyk et al. (2012) conducted a randomized control study comparing video and written education interventions in terms of HPV knowledge and vaccination intentions. The two different formats namely, the pamphlet vs. video on a computer screen, contained identical information proved to be equally effective in improving knowledge and vaccination intentions. Although the current study effectiveness was not assessed, an insight was gained into the public’s communication preferences and the criteria according to which these were formulated. Participants’ preferred mode of communication proved to be the documentary as it provided context to the HPV vaccination, was more, attractive, easier to follow and as such made an impression. Furthermore, participants thought that the documentary did not project an idealized image of the vaccine. Conversely, participants thought that the medical expert was too adamant with regards to the vaccination and this came in sharp contrast to their own experience and did not address their concerns about safety. Evidence in the literature suggests that individuals evaluate stimuli on a given topic according to their own preconceived ideas on that topic despite the content of said stimuli (Peters, 2000). Hence, participants in the present study might have benefited more from a balanced presentation of the HPV vaccination which could address both their knowledge gaps as well as their concerns.

The limitations of this study must also be considered. Real life examples of health communication on HPV were chosen to serve as stimuli over artificially developed ones. Every effort was made to ensure that the basic information on HPV and the HPV vaccine contained in each stimulus was equivalent. However, there was also some variability which might have influenced participants’ judgments of the stimuli. For example, the leaflet contained information on the role of men in HPV transmission, an issue not covered in the other two stimuli. By the same token, the mass vaccination was unique to the documentary, etc.

This study’s findings on the knowledge, beliefs and communication preferences provided an insight on the ways these three critical segments of the Greek public perceive the HPV vaccine as well as the factors that might influence their decision-making process. Health policy adjustments are greatly needed and should be focusing on ensuring that the public has access to appropriate and reliable sources of information in order to make an informed HPV vaccination decision. Specifically, (a) further research should be conducted on the physicians’ role in the communication of health information and support should be provided in the form of guidance, information or resources (b) health information provided by the media should be based on well researched medical facts and presented by science journalists or science communication experts taking into account factors such as format, message framing and prevalent beliefs, (c) the public’s concerns should be investigated further through research and addressed via targeted public health campaigns and (d) sexual health education should be given special focus at schools, local communities and health care contexts. In conclusion, some of the study’s findings might be relevant for countries which have also adopted ‘on demand’ HPV vaccination schemes or are currently considering an HPV vaccination policy.
